# Adjuvant radiotherapy for patients with pathologic node‐negative esophageal carcinoma: A population based propensity matching analysis

**DOI:** 10.1111/1759-7714.13235

**Published:** 2019-12-11

**Authors:** Hui‐Jiang Gao, Xiao‐Bin Shang, Lei Gong, Hong‐Dian Zhang, Peng Ren, Guo‐Dong Shi, Yu‐Cheng Wei, Zhen‐Tao Yu

**Affiliations:** ^1^ Department of Esophageal Cancer, Tianjin's Clinical Research Center for Cancer and Key Laboratory of Cancer Prevention and Therapy Tianjin Medical University Cancer Institute and Hospital Tianjin China; ^2^ Department of Thoracic Surgery The Affiliated Hospital of Qingdao University Qingdao China

**Keywords:** Adjuvant radiotherapy, esophageal carcinoma, esophagectomy, overall survival, SEER database

## Abstract

**Background:**

The impact of adjuvant treatment for esophageal carcinoma with tumor‐negative lymph nodes after upfront radical esophagectomy is still uncertain. This study investigated the effects of postoperative radiotherapy in pT1‐3N0 esophageal carcinoma after radical resection.

**Method:**

We retrospectively identified pT1‐3N0M0 esophageal carcinoma patients between 2000 and 2016 from the Surveillance, Epidemiology, and End Results database. Patients with upfront esophagectomy were categorized as having received surgery alone (SA) and surgical resection followed by adjuvant radiotherapy (SA + RT). Propensity score matching, univariate and multivariate analysis were performed to compare overall survival (OS) and cause‐specific survival (CSS).

**Results:**

A total of 2862 patients were identified, of whom 274 received SA + RT and 2588 received SA. The median follow‐up was 60.4 months (95%CI, 58.7–62.1 months). The five‐year OS and CSS were better for SA group compared with SA + RT group (*P* < 0.001, respectively). Furthermore, after matching, the OS and CSS were still significantly better for SA patients. For T subgroup analysis, postoperative radiotherapy was an independent prognostic factor only for pT1 patients with worse OS, without survival differences for pT2 and pT3 patients. However, after multivariate cox analysis, postoperative radiotherapy can provide significantly better OS for pT3 patients with tumor length ≥5 cm (*P* = 0.03; 95%CI, 0.29–0.94).

**Conclusions:**

Among pT1‐3N0M0 esophageal carcinoma patients, postoperative radiotherapy can provide significantly better OS for pT3 patients with tumor length ≥5 cm. However, there are no survival benefits for pT1‐2 patients after SA + RT procedure. This finding may have significant implications on the use of adjuvant radiation in patients with pN0 disease.

## Introduction

Esophageal carcinoma (EC) is a significant worldwide health problem with poor outcomes. According to the American Cancer Society, an estimated 17 290 people were reported and 15 850 people eventually died of EC in 2018.^1^ Patients frequently present with advanced stage disease at first diagnosis, and its overall prognosis remains compromised, even with multimodality interventions.^2^

Nowadays, several large clinical trials and meta‐analysis with high‐level evidence have confirmed significantly improved oncological outcomes in patients with locally advanced esophageal carcinoma using neoadjuvant chemoradiotherapy (nCRT) followed by surgical resection.^3^, ^4^, ^5^, ^6^ Based on these studies, nCRT plus surgery has become the standard treatment procedure for clinical T1bN1‐N3 or T2‐T4aN−/+M0 patients. Although the use of nCRT has dramatically improved the prognosis for esophageal carcinoma patients, approximately 20% of patients will still develop locoregional recurrence with radical esophageal resection after nCRT,^7^, ^8^, ^9^, ^10^ and the rate of locoregional recurrence has been reported to be up to 42% with surgery alone.^11^, ^12^ Theoretically, adjuvant postoperative therapy can reduce the rate of disease recurrence and improve survival by further eliminating the potential residual tumor and metastatic lymph nodes.^13^, ^14^ Moreover, several clinical trials have shown that adjuvant therapy was associated with improved survival for patients with pathologic node positivity, even after nCRT.^15^, ^16^, ^17^, ^18^ However, the impact of adjuvant treatment for esophageal carcinoma patients who undergo upfront radical esophagectomy is still controversial.

In this study, we analyzed the Surveillance, Epidemiology, and End Results (SEER) database to determine the impact on overall survival (OS) and cause‐specific survival (CSS) for postoperative radiotherapy (postop RT) after upfront radical esophagectomy in patients with pT1‐3N0 esophageal carcinoma.

## Methods

This study was a retrospective analysis of esophageal carcinoma patients recorded in the SEER database, a population‐based cancer registry system collecting data from 18 registries among 14 states across the US, representing nearly 30% of the US population.^19^ Data tracked by SEER include patient demographics, disease characteristics, treatment, and outcome information. Data for all esophageal carcinoma patients from 2000 to 2016 (*n* = 64 625) were acquired in plain text format from SEER and imported into SPSS software version 23.0 (IBM, Armonk, NY, USA) using modified versions of SEER database provided scripts. The endpoints of this current study included OS and CSS, which was the interval between the initial diagnosis of EC and the occurrence of EC‐specific death.

**Figure 1:**
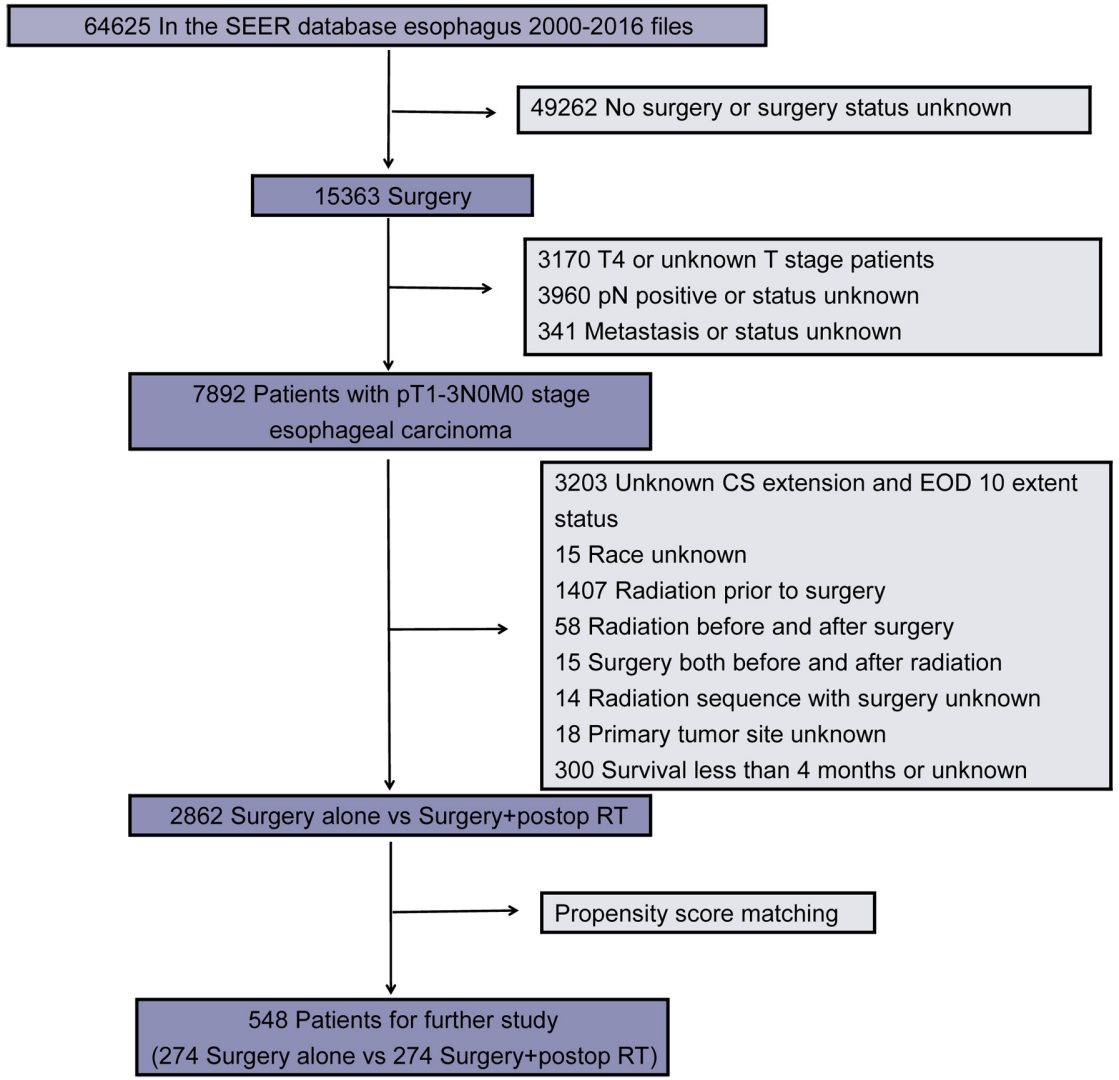
Flow chart for inclusion and exclusion of patients in this study.

We identified patients diagnosed with pT1‐3N0M0 esophageal carcinoma from 2000 to 2016 within the SEER database. Tumor node metastasis (TNM) staging was identified according to the sixth edition American Joint Committee on Cancer (AJCC) TNM system. The inclusion and exclusion criteria are summarized in Fig 1. The inclusion criteria for data extraction in this study were (i) Patients aged older than 18 years and diagnosed with EC between January 2000 to December 2016; and (ii) patients who underwent an esophagectomy alone or postop RT after surgery, with or without partial/total gastrectomies or laryngectomies. The exclusion criteria included (i) Patients confirmed to have pT4 status, positive lymph node involvement at pathological diagnosis, unknown or positive metastatic status; and (ii) patients with missing or incomplete data such as survival status and time, race, T stage, N stage, primary tumor site, pathological type, local treatment, and radiotherapy, together with those who received neoadjuvant therapy or unknown treatment sequence with respect to surgery. In an effort not to exclude patients who received different doses of radiotherapy, the total dose of radiation was not limited.^20^ Since the data from SEER did not include any information which identified the patients, approval of the institutional review board was not required. Furthermore, those who survived ≤four months were also excluded to reduce a bias favoring the postop RT group because some of the patients identified as having received surgery alone may have died in the perioperative period.

## Statistical analysis

Statistical analysis was performed using SPSS software version 23.0 (IBM, Armonk, NY, USA). Mean and standard deviations were used for continuous variables, whereas percentages were used for discrete characteristics. Propensity score matching (PSM) was used to eliminate baseline demographic differences and to achieve better patient group homogeneity using a logistic regression model.^21^ PSM was performed with the following variables: age, sex (male or female), race, disease site, tumor length, histology (squamous or adenocarcinoma), histologic grade, pathological T stage and ELN count. Surgery alone (SA) or surgery followed by postop RT (SA + RT) pairs with a nearest propensity score were matched one to one with a caliper width of 0.2‐fold of standard deviation, and an algorithm was used to sequentially match the next best pair (Figures S1 and S2). Kaplan‐Meier survival analysis and log‐rank test were used for the distributions of OS and CSS. Multivariable analysis were performed using the Cox's proportional hazards regression model. A *P*‐value <0.05 was considered statistically significant.

## Results

**Figure 2:**
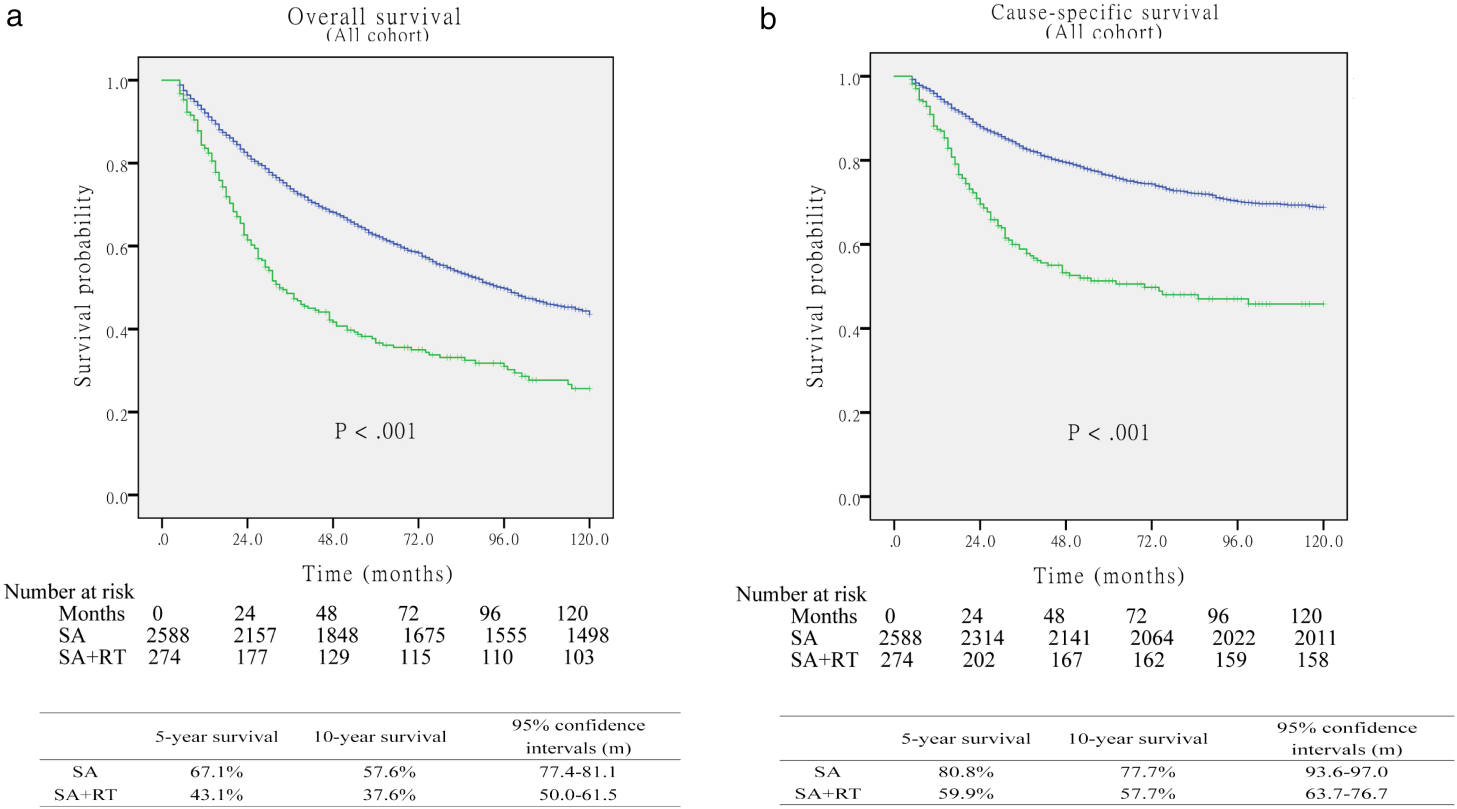
(**a**) Overall survival between surgery alone and surgery+postoperative RT (postop RT) groups before matching (*P* < 0.001). (**b**) Cause‐specific survival between surgery alone and surgery+postoperative RT groups before matching (*P* < 0.001). 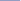
surgery alone, 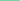
surgery+postop RT, 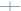
SA‐censored, 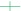
SA+RT‐censored.

**Figure 3:**
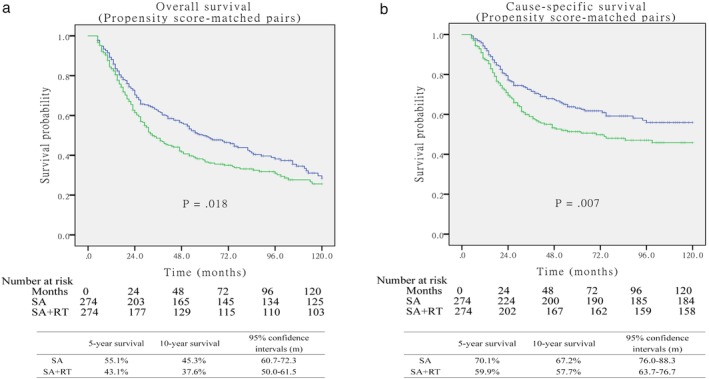
(**a**) Overall survival between surgery alone and surgery+postoperative RT (postop RT) groups after matching (*P* = 0.018). (**b**) Cause‐specific survival between surgery alone and surgery+postop RT groups after matching (*P* = 0.007). 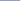
surgery alone, 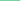
surgery+postop RT, 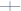
SA‐censored, 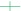
SA+RT‐censored.

The characteristics of the 2862 patients before PSM are summarized in Table 1, of whom 274 patients received surgery followed by postoperative radiotherapy and 2588 patients received surgery alone. The median follow‐up period after surgery was 60.4 months (95% CI, 58.7–62.1 months). Patients in the SA group were more likely to have a significantly smaller total tumor size, earlier pT stage, and better differentiated histologic grade. Among all patients, those who received the SA procedure showed significantly better OS and CSS (*P* < 0.001, respectively) when compared to patients in the SA + RT group (Fig 2). After propensity score matching, 274 patients in the SA group were matched and compared with 274 patients in the SA + RT group. Variables were included without significant differences in demographic factors (Table 2). Taking into account all matched patients, the OS and CSS were still significantly better for SA patients (*P* = 0.018 and *P* = 0.007, respectively; Fig 3).

**Table 1:** Baseline characteristics of patients

Characteristics	Surgery alone (*n* = 2588)	Surgery+postop RT (*n* = 274)	*P*‐value
Age, years ± SD	66.4 ± 10.3	65.6 ± 10.6	0.27
Male sex, *n* (%)	2066 (79.8)	220 (80.3)	0.86
Race/ethnicity, *n* (%)			0.28
White	2348 (95.3)	243 (88.7)	
Other	240 (4.7)	31 (11.3)	
Disease site, *n* (%)			0.69
Upper third	113 (4.4)	15 (5.5)	
Middle third	491 (18.9)	50 (18.2)	
Lower third	1984 (79.7)	209 (76.3)	
Tumor length, cm, *n* (%)			<0.001
<3	1709 (66.1)	120 (43.8)	
3–5	524 (20.2)	82 (29.9)	
≥5	355 (13.7)	72 (26.3)	
Tumor histology, *n* (%)			0.02
Squamous cell carcinoma	523 (20.2)	73 (26.6)	
Adenocarcinoma	1890 (73.0)	179 (65.3)	
Other	175 (6.8)	22 (8.1)	
Histologic grade, *n* (%)			<0.001
Well	704 (27.2)	46 (16.8)	
Moderate	1173 (45.3)	106 (38.7)	
Poor	666 (25.7)	120 (43.8)	
Undifferentiated	45 (1.8)	2 (0.7)	
Pathological T stage, *n* (%)			<0.001
1	1895 (73.2)	101 (36.9)	
2	368 (14.2)	61 (22.2)	
3	325 (12.6)	112 (40.9)	
ELN count, *n* ± SD	12.2 ± 17.2	11.5 ± 20.0	0.48

ELN, examined lymph node; postop RT, postoperative radiation therapy.

**Table 2:** Characteristics for patients with pT1‐3N0 esophageal carcinoma after propensity score matching (PSM)

			Standardized difference	
Characteristics	Surgery alone (*n* = 274)	Surgery+postop RT (*n* = 274)	Before	After
Age, years ± SD	66.2 ± 10.4	65.6 ± 10.6	−0.069	−0.022
Male sex, *n* (%)	220 (80.3)	220 (80.3)	−0.012	0.000
Race/ethnicity, *n* (%)			−0.064	0.000
White	243 (88.7)	243 (88.7)		
Other	31 (11.3)	31 (11.3)		
Disease site, *n* (%)			−0.019	0.038
Upper third	17 (6.2)	15 (5.5)		
Middle third	46 (16.8)	50 (18.2)		
Lower third	221 (80.7)	209 (76.3)		
Tumor length, cm, *n* (%)			0.211	−0.040
<3	126 (45.9)	120 (43.8)		
3–5	87 (31.8)	82 (29.9)		
≥5	61 (22.3)	72 (26.3)		
Tumor histology, *n* (%)			−0.162	0.008
Squamous cell carcinoma	80 (29.2)	73 (26.7)		
Adenocarcinoma	178 (64.9)	179 (65.3)		
Other	16 (5.8)	22 (8.0)		
Histologic grade, *n* (%)			−0.136	−0.007
Well	40 (14.6)	46 (16.8)		
Moderate	107 (39.1)	106 (38.7)		
Poor	125 (45.6)	120 (43.8)		
Undifferentiated	2 (0.7)	2 (0.7)		
Pathological T stage, *n* (%)			0.193	−0.096
1	99 (36.1)	101 (36.9)		
2	72 (26.3)	61 (22.3)		
3	103 (37.6)	112 (40.8)		
ELN count, *n* ± SD	10.9 ± 11.9	11.5 ± 20.0	−0.040	0.029

ELN, examined lymph node; postop RT, postoperative radiation therapy.

**Figure 4:**
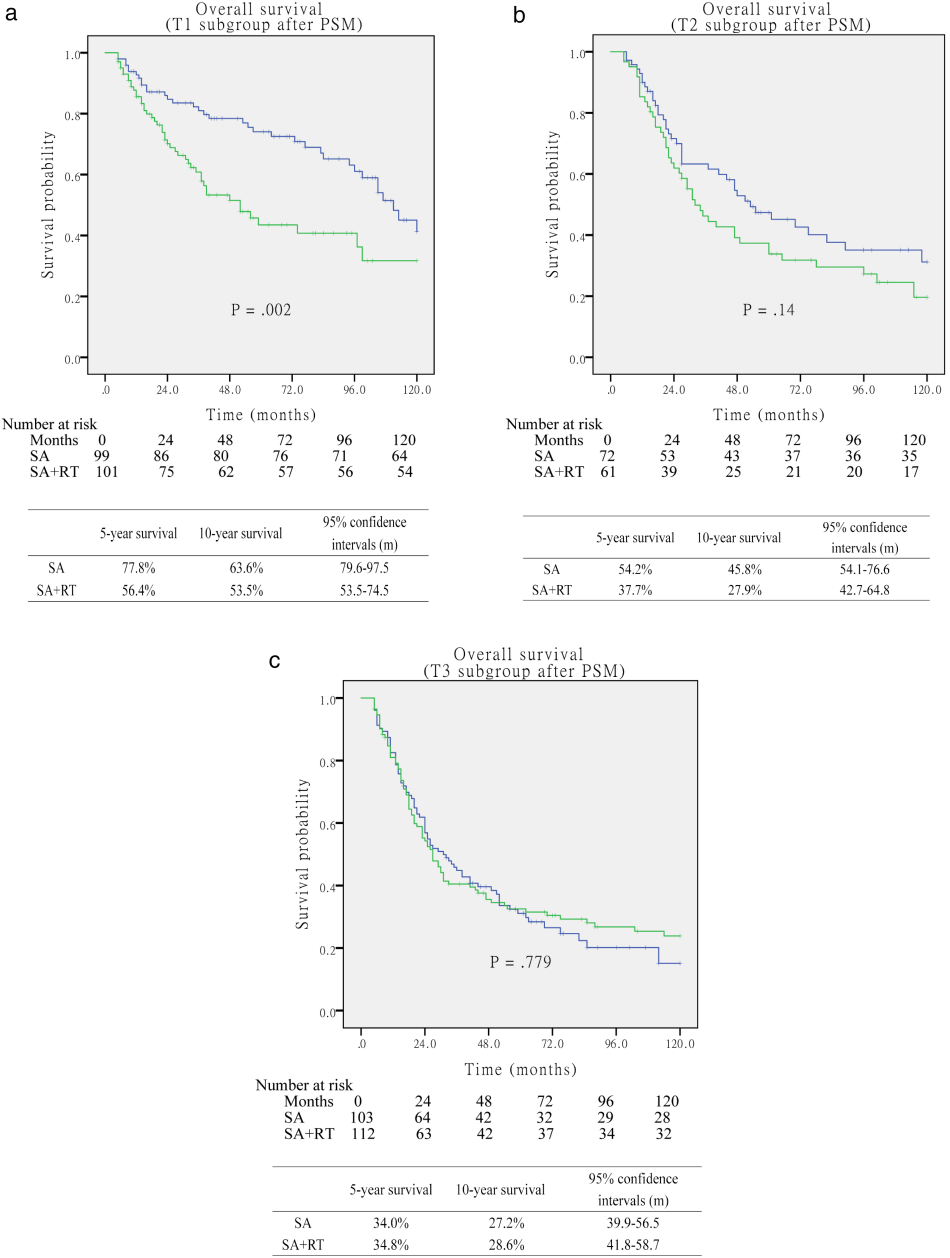
(**a**) Overall survival between surgery alone and surgery+postoperative RT(postop RT) groups with pT1 subgroup (*P* = 0.002). (**b**) Overall survival between surgery alone and surgery+postop RT groups with pT2 subgroup (*P* = 0.14). (**c**) Overall survival between surgery alone and surgery+postop RT groups with pT3 subgroup (*P* = 0.779). 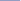
surgery alone, 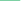
surgery+postop RT, 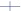
SA‐censored, 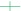
SA+RT‐censored.

The multivariable cox analysis for OS with or without postop RT when stratified by T subgroups after PSM are described in Table 3. Postop RT was an independent prognostic factor only for T1 patients with worse OS (*P* = 0.004). However, no differences were noted in the pT2 and pT3 subgroups for SA compared to the SA + RT approach (*P* = 0.21 and *P* = 0.47, respectively). After matching, pT3 patients in the SA + RT group seemed to have a better survival outcome; however, no significant differences were observed in OS between patients in the SA group and SA + RT group (*P* = 0.779; Fig 4).

**Table 3:** Multivariable cox analysis for OS with or without postoperative RT (postop RT) when stratified by pT subgroups after propensity score matching (PSM)

			Multivariable analysis	
Cohort	Surgery alone five‐year OS (%)	Surgery+postop RT five‐year OS (%)	HR (95% CI)	*P*‐value
Overall cohort	55.1	43.4	0.66 (0.52–0.84)	0.001
T1	77.8	56.4	0.45 (0.27–0.78)	0.004
T2	54.2	37.7	0.73 (0.45–1.19)	0.21
T3	34.0	34.8	0.88 (0.61–1.25)	0.47

postop RT, postoperative radiation therapy.

In the univariable and multivariable analysis for all‐cause mortality, according to pT3 subgroup characteristics, no significant differences in OS were observed on SA and SA + RT procedures in the subgroups for age, sex, race/ethnicity, disease site, histology and histologic grade status (Table 4). Patients who underwent the SA + RT procedure showed significantly better OS compared to patients who underwent the SA procedure in the subgroup with tumor length ≥5 cm (*P* = 0.03; 95% CI, 0.29–0.94); however, no differences were observed in the subgroup with tumor length <5 cm (*P* = 0.25; 95% CI, 0.83–2.01). Besides, examined lymph node (ELN) count ≥14 was demonstrated as an independent factor for worse OS regardless of tumor size or other variables included in this study (*P* = 0.04; 95% CI, 0.28–0.98). Similarly, ELN count <13 was not an independent favorable prognostic factor for OS after multivariate cox analysis (*P* = 0.18; 95% CI, 0.87–2.06).

**Table 4:** Univariable and multivariable hazard ratios for all‐cause mortality, according to pT3 subgroup characteristics

			Univariable analysis		Multivariable analysis	
Cohort	Surgery alone (*n* = 103)	Surgery+postop RT (*n* = 112)	HR (95% CI)	*P*‐value	HR (95% CI)	*P*‐value
Age, *n* (%)						
≤60	31 (30.1)	41 (36.6)	1.13 (0.65–1.97)	0.67	1.01 (0.52–1.95)	0.97
> 60	72 (69.9)	71 (63.4)	0.99 (0.67–1·45)	0.94	1.02 (0.67–1.54)	0.94
Sex, *n* (%)						
Male	80 (77.7)	90 (80.4)	1.02 (0.71–1·45)	0.93	0.96 (0.66–1.41)	0.85
Female	23 (22.3)	22 (19.6)	1.15 (0.56–2.39)	0.71	1.40 (0.51–3.89)	0.52
Race/ethnicity, *n* (%)						
White	87 (84.5)	94 (83.9)	1.09 (0.77–1·55)	0.62	0.97 (0.67–1.41)	0.87
Other	16 (15.5)	18 (16.1)	0.91 (0.40–2.05)	0.81	1.08 (0.36–3.21)	0.89
Disease site, *n* (%)						
Upper third	8 (7.8)	5 (4.5)	0.84 (0.24–2.93)	0.79	0.33 (0.03–4.49)	0.41
Middle third	17 (16.5)	27 (24.1)	0.62 (0.31–1.23)	0.17	0.59 (0.22–1.59)	0.30
Lower third	78 (75.7)	80 (71.4)	1.25 (0.85–1.83)	0.26	1.16 (0.77–1.75)	0.48
Tumor length, *n* (%)						
<5 cm	67 (65.1)	68 (60.7)	0.65 (0.41–1.05)	0.08	1.29 (0.83–2.01)	0.25
≥5 cm	36 (34.9)	44 (39.3)	0.69 (0.39–1.19)	0.18	0.52 (0.29–0.94)	0.03
Histology, *n* (%)						
SCC	41 (39.8)	38 (33.9)	0.66 (0.39–1.09)	0.11	0.76 (0.41–1.43)	0.39
Adenocarcinoma	52 (50.5)	67 (59.8)	1.73 (1.11–2.67)	0.01	1.47 (0.93–2.33)	0.09
Histologic grade, *n* (%)						
Well+moderate	43 (41.7)	55 (49.1)	1.28 (0.79–2.07)	0.31	1.49 (0.85–2.61)	0.17
Poor	60 (58.3)	57 (50.9)	0.83 (0.54–1.27)	0.39	0.76 (0.48–1.22)	0.26
ELN count, *n* (%)						
<13	65 (63.1)	67 (59.8)	0.79 (0.48–1.32)	0.37	1.34 (0.87–2.06)	0.18
≥14	37 (35.9)	45 (40.2)	0.71 (0.42–1.22)	0.22	0.53 (0.28–0.98)	0.04

ELN, examined lymph node; postop RT, postoperative radiation therapy; SCC, squamous cell carcinoma.

## Discussion

Neoadjuvant chemoradiation for esophageal carcinoma has been previously tested in several randomized controlled trials with beneficial oncological outcomes, thus establishing this approach as the standard of care for locoregional advanced patients prior to surgery.^3^, ^4^, ^5^, ^6^ However, the optimal postoperative management of patients who undergo upfront esophagectomy is still unclear. The national guidelines currently recommend consideration of postoperative adjuvant chemoRT for patients with locally advanced (T2‐T4N0‐1) adenocarcinoma or patients with incomplete resection, but these lack high level evidence.

Several large clinical trials have evaluated the role of postoperative radiotherapy (postop RT) but have reported conflicting results. Fok *et al*. randomized 130 patients with either esophageal squamous cell carcinoma or adenocarcinoma to either postop RT or observation after upfront esophagectomy. Although postop RT significantly improved local recurrence control (20% with vs. 46% without postop RT), the trimodality procedure was associated with a decrease in OS (median survival 8.7 with vs. 15.2 months without postop RT), probably due to an increase in treatment‐related deaths.^22^ Besides, another meta‐analysis carried out by Malthaner *et al*., which included the aforementioned trial, also failed to detect a significant survival difference with the use of postop RT after upfront esophagectomy (*P* = 0.11; 95% CI, 0.95–1.59).^23^ However, the limitations of those patients with advanced disease have not been adequately powered to stratify outcomes by disease stage.

A recent study using the SEER database has shown survival benefits of postop RT for patients with clinical stage III disease (T3N1M0 or T4N0‐1M0, AJCC sixth edition), although this study included patients with squamous cell or adenocarcinoma.^24^ Another single institution series study also found that postop RT was associated with better OS for esophageal squamous cell carcinoma patients with node‐positive disease, stage III/IV, and large or deeply invasive tumors.^25^ Therefore, those population‐based analysis demonstrated a significant survival benefit from the addition of postop RT for patients with node‐positive disease.

For tumor‐negative lymph nodes patients after upfront radical esophagectomy, the optimal treatment procedure is still uncertain. Although postop RT has been widely used, only a few studies have been designed to specifically validate the potential benefits of postop RT for upfront radical esophagectomy with pathologically‐negative lymph nodes. The aforementioned studies recommended no further treatment for node negative patients who underwent upfront radical resection.^22^, ^23^, ^24^, ^25^ However, many of these studies included patients with both tumor‐negative and positive lymph nodes. For example, one of these trials driven from NCDB, included patients with with stage pT3‐4Nx‐0 or pT1‐4N1‐3 esophageal carcinoma (squamous cell or adenocarcinoma) without metastatic disease.^16^ This hospital‐based study showed improved long‐term oncologic benefits for patients treated with postop RT compared to surgery alone. However, when survival outcomes were analyzed by node status, postop RT appeared to be no significant benefit for tumor‐negative lymph nodes patients than that of positive patients. Another single institution series of 692 patients with radically resected T3 esophageal squamous cell carcinoma (246 received postop RT) by Chen *et al*. found that postop RT was associated with better local control and OS for patients with tumor length >5 cm, pN0 and pN1 categories, pTNM stage IIa/IIb/IIIa may be improved.^26^ Therefore, there is also a paucity of data regarding which patient population among upfront esophagectomy and node negative esophageal carcinomas derives more benefit from postoperative therapy.

In this large hospital‐based study, unlike previous analysis, tumor‐negative lymph nodes status was only assessed in the context of postop RT usage. We noted that the use of esophagectomy alone was associated with a 24% absolute five‐year OS benefit compared with postop RT after esophagectomy. Even after PSM, there was still a 12% absolute OS benefit for the SA group compared with the SA + RT group. On subgroup analysis, this disadvantage finding for postop RT was driven by patients with pT1 disease, rather than pT2 and pT3 diseases. The pT stage is an important indicator for evaluating the prognosis of patients with esophageal carcinoma. For patients with pT3 disease, there was only an 0.8% absolute improvement in five‐year OS with the use of postop RT compared with surgery alone from 34.8% to 34% without significant difference (*P* = 0.779). Comparably, patients with pT1‐2 disease had a disadvantage improvement in five‐year OS with postop RT (21.4% and 16.5%, respectively). There are few other studies which have specifically investigated the role of postop RT for patients with N0 status after upfront esophagectomy. A retrospective, SEER database by Shridhar *et al*. included 2109 patients with esophageal carcinoma who underwent esophagectomy and postop RT, and 1373 of these patients had negative lymph nodes. They reported postop RT was associated with decreased survival in node negative patients, and there was no relationship between pT stage and survival outcomes.^27^ Thus, our study suggest that a significant disadvantage in survival can be attained by pT1 status with postop RT. However, there was no significant survival difference for pT2 and pT3 patients with, or without, postop RT.

Although not included in the TNM staging system for esophageal carcinoma, the variable of tumor length has been demonstrated to be an independent prognostic factor for survival in several studies.^26,28‐–30^ Semenkovich *et al*. developed a decision analysis model which depended upon a total of 4013 cT2N0 esophageal carcinoma patients from 10 relevant studies. Tumor length larger than 3 cm was one of the key variables associated with a >48.1% risk of upstaging.^28^ Shridhar *et al*. analyzed a total of 1840 patients from the NCDB between 2004 to 2013 and showed that tumor length > 3 cm and poor differentiation were significantly associated with tumor upstaging.^29^ Another retrospective study basing on NCDB for patients with T1a esophageal adenocarcinoma who underwent esophagectomy or endoscopic resection and generated a balanced cohort with 735 matched pairs using propensity‐score matching also confirmed that tumor length was one of the most important risk factors for nodal metastases.^30^ Owing to the aforementioned studies, tumor length is an important risk factor thus conferring a survival advantage to induction and/or postoperative adjuvant therapy. In our study, however, multivariate analysis showed that postop RT was not an independent risk factor for prognosis of pT3 patients. However, in the univariable and multivariable analysis for all‐cause mortality, according to pT3 subgroup characteristics, postop RT was an independent factor for better OS compared to patients who underwent SA procedure in the subgroup with tumor length ≥5 cm.

The majority of patients included in this study had esophageal adenocarcinoma. As the incidence of esophageal adenocarcinoma continues to rise rapidly in the United States, studies specifically examining the role of adjuvant radiotherapy in this subgroup are increasingly relevant.^31^ One of the large randomized trials by Xiao *et al*. included only patients with squamous cell carcinoma, so the findings in that trial may not necessarily be representative of patients with adenocarcinoma.^23^ Another retrospective study by Wong *et al*. including adenocarcinoma and squamous cell carcinoma patients found that histologic subtype did not have a significant impact on survival on multivariate analysis after propensity score matching.^16^ Although some data suggest that squamous cell carcinomas may be more responsive to chemoRT than adenocarcinomas,^4^ all these studies failed to focus the tumor histology on pathological node‐negative patients. In our study, multivariate analysis found that histologic subtype did not have a significant impact on survival, even after propensity score matching.

To our knowledge, this is the largest study to date to specifically examine the role of postop RT after esophagectomy in pN0 patients. However, several limitations inherent to the SEER database itself should be noted. First, our results were obtained by a retrospective analysis. The patients were grouped based on treatment mode (SA vs. SA + RT) and were thus not randomized, potentially resulting in a selection bias. Second, no information was recorded on radiation technologies in the SEER database. This aspect requires further investigation given that radiation therapy technology has rapidly evolved over the past few decades and survival rates are dependent on the radiation technologies used. Additionally, some variables were not included in this database, such as R0 resection (including the circumferential margin), patient comorbidities, performance status, lymphovascular invasion, type of lymphadenectomy and gene mutations, which make it difficult to ascertain whether the survival benefits should be attributed to these unmeasured confounders.

In conclusion, this large, hospital‐based analysis demonstrated a significant survival benefit from the addition of postop RT for node‐negative pT3 patients in the subgroup with tumor length ≥5 cm. However, there was no survival benefit for pT1‐2 patients after postop RT. Although unmeasured confounders within the SEER database limit the conclusions one can draw from this analysis, these data do suggest that further studies may be needed to identify the potential roles of postop RT in the setting of tumor‐negative lymph nodes status.

## Disclosure

The authors have no conflict of interest.

## Supporting information


**Figure S1** Histogram of propensity scores for patients between the surgery alone group and surgery+postop RT group. (**a**) unmatched patients who received surgery alone. (**b**) matched patients who received surgery alone. (**c**) unmatched patients who received surgery+postop RT. (**d**) matched patients who received surgery+postop RT. Matched groups have similar propensity score distributions.

**Figure S2** Standardized differences of variables between patients who received surgery alone and those who received surgery+postop RT. Hollow diamond symbolized differences before propensity matching and black diamond symbolized differences after propensity matching. Propensity matching effectively reduced heterogeneity among variables between the two surgical approaches in comparison (ELN, examined lymph node).
