# Supplementary figures and images for: Adjuvant radiotherapy for patients with pathologic node‐negative esophageal carcinoma: A population based propensity matching analysis
Source: Thorac Cancer. 2019 Dec 11;11(2):243–52. doi: 10.1111/1759-7714.13235 (PMC6996980; doi:10.1111/1759-7714.13235)

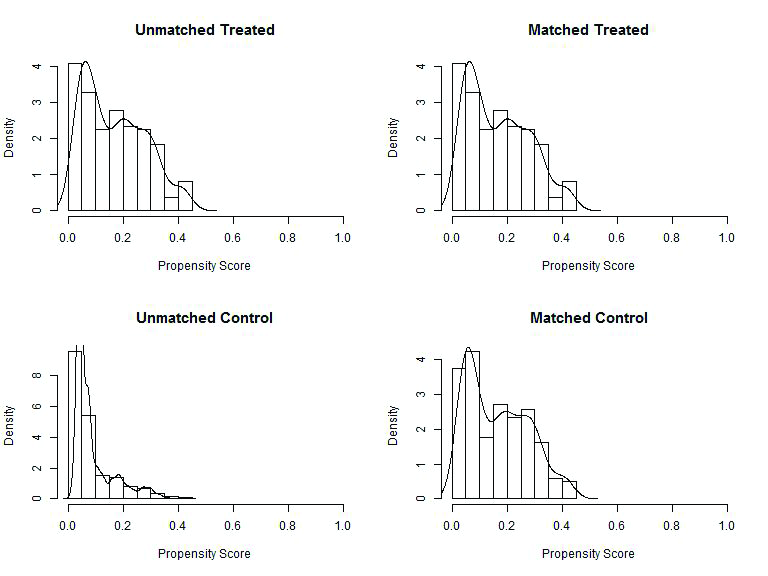

Supplement: Supplementary file 1 — Figure S1 Histogram of propensity scores for patients between the surgery alone group and surgery+postop RT group. (a) unmatched patients who received surgery alone. (b) matched patients who received surgery alone. (c) unmatched patients who received surgery+postop RT. (d) matched patients who received surgery+postop RT. Matched groups have similar propensity score distributions. [file TCA-11-243-s001.tif]

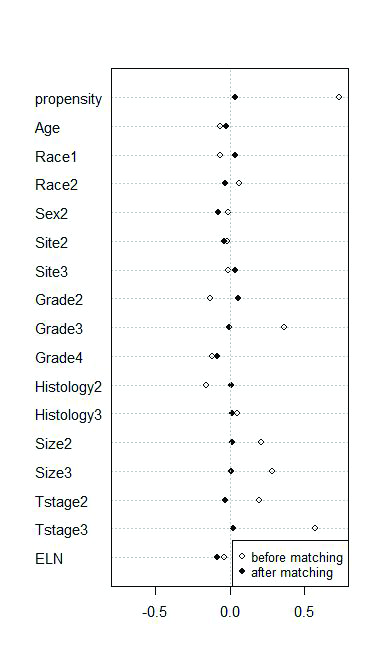

Supplement: Supplementary file 2 — Figure S2 Standardized differences of variables between patients who received surgery alone and those who received surgery+postop RT. Hollow diamond symbolized differences before propensity matching and black diamond symbolized differences after propensity matching. Propensity matching effectively reduced heterogeneity among variables between the two surgical approaches in comparison (ELN, examined lymph node). [file TCA-11-243-s002.tif]
